# The Multifaceted Aspects of Oxidative Stress in the Skin and Other Tissues

**DOI:** 10.3390/antiox13091081

**Published:** 2024-09-04

**Authors:** Reinhart Speeckaert, Marijn M. Speeckaert, Nanja van Geel

**Affiliations:** 1 Department of Dermatology, Ghent University Hospital, Corneel Heymanslaan 10, 9000 Ghent, Belgium; nanja.vangeel@ugent.be; 2 Department of Nephrology, Ghent University Hospital, Corneel Heymanslaan 10, 9000 Ghent, Belgium; marijn.speeckaert@ugent.be

Different tissues experience various levels of oxidative stress based on their function and protection from outside environments. The skin is particularly vulnerable to outside triggers due to its complex environmental interface which protects internal tissues from chemicals, pollutants, and allergens to maintain body homeostasis. Many external pollutants are oxidants, or directly or indirectly increase the production of reactive oxygen species (ROS). Their key role is not only to combat invading pathogens and degrade damaged tissues, but also to regulate genes involving ion channels, modulate transcription factors, modify the phosphorylation and dephosphorylation of enzymes, and influence epigenetic modifications [1,2]. However, when chronic or acute oxidative factors overwhelm the antioxidative defense mechanisms, ROS also trigger inflammatory, proliferative, and apoptotic pathways leading to many skin disorders [3]. Stressed keratinocytes can produce a large range of chemokines including the C-X-C motif chemokine ligand (CXCL)1/2/3/4/8 which recruits neutrophils. Neutrophils generate additional ROS, leading to further epidermal changes [4].

In cases where the skin barrier becomes impaired, such as in atopic dermatitis (AD), environmental insults have an even greater impact. The resulting inflammation decreases structural proteins and tight junction components. The lack of keratinocyte adhesion and the disrupted stratum corneum leads to high transepidermal water loss and skin xerosis. This results in an increased penetration of pathogens and allergens, with subsequent activation of the immune system [5]. The direct involvement of environmental pollutants and UV light in AD is suggested by the correlation of dinitrophenylhydrazone (DNP) levels in the skin with AD severity. These DNP levels, resulting from the reaction of dinitrophenylhydrazine with oxidative stress-induced carbonyl compounds such as malonaldehyde, are notably higher in the superficial layers of the stratum corneum [6]. In this Issue, Ardizzone et al. used tempol as a radical scavenger in an AD mouse model (Contribution 1). In addition to a reduction in oxidative stress markers [nuclear factor erythroid 2-related factor 2 (Nrf2), manganese superoxide dismutase (MnSOD), and heme oxygenase I (HO-1) expression], a decrease in inflammatory cytokines [tumor necrosis factor-alpha (TNF-α), interleukin-1 beta (IL-1β)] and an improvement in histological damage were reported. These findings highlight the potential of topical antioxidants for the treatment of AD. Previously, photoprotective properties of tempol against UV radiation on fibroblasts were also reported [7]. Nonetheless, the overall use of antioxidants in AD remains controversial. A systematic review showed an additional benefit of antioxidants, but only in pediatric AD patients and not in adults. Oral vitamin D and topical vitamin B12 were the most promising antioxidants [8]. The publication of Ardizzone et al. (Contribution 1) is therefore important to further strengthen the evidence around the use of antioxidants in AD.

In wound healing, oxidative stress plays an intriguing dual role. An initial increase in ROS levels is helpful to initiate protection against pathogens and to induce signaling cascades leading to re-epithelialization. The healing of ulcers involves different phases, consisting of inflammation, formation of granulation tissue, angiogenesis, and re-epithelialization [9]. During hemostasis and immune activation, superoxide is generated by immune cells. Redox signaling also regulates cell migration, fibrosis, proliferation, and tissue remodeling. In contrast, excessive ROS concentrations significantly delay wound healing by damaging epidermal cells, prolonging inflammatory responses, lipid peroxidation, protein modification, and DNA damage. These changes result in high cell apoptosis and increased senescence. The delicate balance of oxidative stress should therefore be closely modulated to decrease the time to wound closure. The monitoring of oxidative damage and antioxidative defenses can be used to evaluate the effectivity of tissue repair treatments (Contribution 2).

Although located deeper in the skin and further away from superficial environmental damaging factors at the basal layer, melanocytes are particularly vulnerable to oxidative stress. The process of melanogenesis increases the production of ROS in melanosomes. These specialized organelles protect other cellular components from oxidative damage. When melanocytes become actively targeted for immune destruction in vitiligo, the added inflammatory component contributes to the increased oxidative stress detected in vitiligo patients. Vitiligo melanocytes have been reported to be more susceptible to oxidative stress compared to melanocytes from healthy donors. The impaired activities of antioxidant defenses may actively induce the loss of melanocytes observed in vitiligo [10]. The therapeutic targeting of oxidative stress in vitiligo is, however, challenging. Several antioxidants have been successfully tested, including polypodium leucotomos, ginkgo biloba, catalase/superoxide dismutase, and vitamin E (Contribution 3). Nonetheless, reports confirming this are scarce. Moreover, combination treatments are often used, and adequate comparator groups are sometimes missing, which makes it difficult to draw accurate conclusions on the efficacy of individual compounds. An increase in the minimal erythema dose (MED) resulting in less inflammation after UV exposure may be a key reason why antioxidants are especially effective when combined with phototherapy in vitiligo. The combination of an anti-inflammatory effect with stimulation of melanocytes enhances repigmentation while preventing the immune-mediated destruction of melanocytes, facilitating the repigmentation of vitiligo lesions. As oxidative stress plays a role in melanogenesis, antioxidants are also actively researched in hyperpigmentary disorders, including melasma, and are present in many over-the-counter anti-pigmentation products. Oxidative stress correlates with the disease severity of melasma. Many antioxidants, such as vitamin C, inhibit tyrosinase, which is the crucial enzyme in the conversion of L-tyrosine to melanin and therefore regulates melanogenesis [11]. Tranexamic acid has another working mechanism, blocking the melanosome transfer from keratinocytes to melanocytes [12]. Often, combinations of several antioxidants are used in topical formulations to improve melasma [11]. Sunlight is also an active contributor to oxidative stress, which is an additional argument for strict sun-protective measures in melasma patients.

Hyperpigmentation is not the only disease where UV light is an important inducer of oxidative free radicals. A primary example is lupus, which is the archetypical autoimmune disorder worsened by sunlight and other types of UV exposure. Its involvement goes far beyond the skin, with the kidney being one of its primary targets [13]. Oxidative stress worsens kidney damage in lupus nephritis and drives a vicious circle of inflammation. Chen et al. (Contribution 4) showed the potential of quiercetin, a natural flavonoid present in vegetables, fruit, and herbs, for treatment of lupus nephritis in mouse and in vivo models. The effects were not only noticeable due to a reduction in inflammatory pathways, but also by an improvement in IL-33-induced fibrosis. IL-33 is a crucial cytokine in systemic lupus erythematosus (SLE) patients and has been proposed as a biomarker and therapeutic target. Together with a lower deposition of collagen in the kidney tissue, less vimentin and fibronectin, as well as improved pro-inflammatory cytokines, damage-associated molecular patterns (DAMPs), and inflammasome, the effects of quiercetin seem promising. It should be noted that several studies have already demonstrated the positive effects of quiercertin in different autoimmune disorders including rheumatoid arthritis, inflammatory bowel disease, multiple sclerosis, Graves’ disease AD, and SLE [14]. This strengthens the evidence for the therapeutic use of quiercetin in pathogenic pro-inflammatory processes.

Antioxidants often regulate multiple pathways independent of their antioxidative properties. Interestingly, the effects of quiercetin on IL-33-induced fibrosis were shown in subsequent experiments to be likely unrelated to its antioxidative capacity; rather, they reflect an anti-inflammatory and anti-fibrotic capacity. Li et al. showed in a chronic graft versus host disease mouse model that quiecertin can prevent lupus nephritis by reducing CD4 T cell activation, decreasing the production of autoantibodies, and lowering proinflammatory cytokine levels [15]. The multifactorial working mechanisms of many antioxidants underline a crucial pitfall for clinical trials using antioxidants, as not all effects may be attributed to its free radical scavenging properties. Inflammation and oxidative stress are simultaneously found in many pathological pathways. Both represent closely related processes and one can be easily induced by the other and vice versa. Although research has abundantly shown the triggering effect of oxidative stress on inflammation, the results of human clinical trials using antioxidants have shown mixed and rather disappointing results. This has been termed ‘the antioxidant paradox’. In the context of inflammation, antioxidants may require, in addition to an antioxidative effect, an anti-inflammatory action in order to be clinically effective. While oxidative stress initiates and triggers inflammation, inhibition of this pathway often seems insufficient to dampen highly active inflammatory environments [2].

Given its function in metabolism and detoxification, the liver is subjected to chronic oxidative stress, and increased oxidative stress can exacerbate liver diseases, including hepatitis and fibrosis, by promoting hepatocyte death and fibrogenesis. The liver has a large quantity of mitochondria and ROS are produced during oxidative phosphorylation. Hepatocytes are therefore highly susceptible to oxidative stress [5]. Carbon tetrachloride (CCl_4_) is a potent toxicant which can be used in murine models to study hepatoxicity. In this Special Issue, Alnuqaydan et al. (Contribution 5) showed that Tamarix articulata can decrease pro-inflammatory markers [e.g., TNF-α, IL-6, and transforming growth factor (TGF)-β] and restore antioxidant enzymes leading to the reversal of liver fibrosis. Tamarix articulata contains flavonoids and polyphenols. Its anti-scavenging effect may explain the hepatoprotective activity. The chemical components of Tamarix articulata extract include gallic acid, quinic acid, kaemperfol, quiecertin, tamarixetin, epiafzelechin, and epicatechin gallate. It has several pharmaceutical properties including immunomodulatory, antibacterial, antiviral antidiabetic, and anticancer effects. It also inhibits the proliferation of hepatocellular carcinoma cells and promotes their cell death [16]. The beneficial effects of Tamarix articulata were already reported in other cancer types, such as prostate cancer, by exhibiting antiproliferative and antimetastatic properties via the modulation of phosphoinositide 3-kinase (PI3K)-Akt and TGF-β signaling [17]. The research on Tamarix articulata extends to the field of dermatology. It offers protection against H_2_0_2_-medicated oxidative stress in skin fibroblasts and counters the cytotoxicity and oxidative stress-mediated apoptosis of heavy metals present in beauty products [18,19].

Overall, understanding the roles of oxidative stress and antioxidants across various diseases highlights the complexity of this biochemical process and its intriguing interplay with other pathogenetic events. Human tissues have different exposures to environmental factors and experience various amounts of oxidative stress due to their physiological function. New research, such as that published in this Special Issue, offers promising insights that help to improve outcomes across a broad spectrum of medical disciplines.
